# Effect of a Low-Carbohydrate High-Fat Diet and a Single Bout of Exercise on Glucose Tolerance, Lipid Profile and Endothelial Function in Normal Weight Young Healthy Females

**DOI:** 10.3389/fphys.2019.01499

**Published:** 2019-12-19

**Authors:** Thorhildur Ditta Valsdottir, Christine Henriksen, Nancy Odden, Birgitte Nellemann, Per B. Jeppesen, Jonny Hisdal, Ane C. Westerberg, Jørgen Jensen

**Affiliations:** ^1^Department of Medicine, Atlantis Medical University College, Oslo, Norway; ^2^Department of Physical Performance, Norwegian School of Sport Sciences, Oslo, Norway; ^3^Department of Nutrition, Institute of Basic Medical Sciences, Faculty of Medicine, University of Oslo, Oslo, Norway; ^4^Department of Nutrition, Atlantis Medical University College, Oslo, Norway; ^5^Department of Clinical Medicine, Aarhus University, Aarhus, Denmark; ^6^Oslo Vascular Center, Department of Vascular Surgery, Oslo University Hospital, Oslo, Norway; ^7^Institute of Health Sciences, Kristiania University College, Oslo, Norway

**Keywords:** low-carbohydrate diet, high-fat diet, glucose tolerance, cholesterol, exercise, suPAR, flow-mediated dilation

## Abstract

Low-carbohydrate-high-fat (LCHF) diets are efficient for weight loss, and are also used by healthy people to maintain bodyweight. The main aim of this study was to investigate the effect of 3-week energy-balanced LCHF-diet, with >75 percentage energy (E%) from fat, on glucose tolerance and lipid profile in normal weight, young, healthy women. The second aim of the study was to investigate if a bout of exercise would prevent any negative effect of LCHF-diet on glucose tolerance. Seventeen females participated, age 23.5 ± 0.5 years; body mass index 21.0 ± 0.4 kg/m^2^, with a mean dietary intake of 78 ± 1 E% fat, 19 ± 1 E% protein and 3 ± 0 E% carbohydrates. Measurements were performed at baseline and post-intervention. Fasting glucose decreased from 4.7 ± 0.1 to 4.4 mmol/L (*p* < 0.001) during the dietary intervention whereas fasting insulin was unaffected. Glucose area under the curve (AUC) and insulin AUC did not change during an OGTT after the intervention. Before the intervention, a bout of aerobic exercise reduced fasting glucose (4.4 ± 0.1 mmol/L, *p* < 0.001) and glucose AUC (739 ± 41 to 661 ± 25, *p* = 0.008) during OGTT the following morning. After the intervention, exercise did not reduce fasting glucose the following morning, and glucose AUC during an OGTT increased compared to the day before (789 ± 43 to 889 ± 40 mmol/L∙120min^–1^, *p* = 0.001). AUC for insulin was unaffected. The dietary intervention increased total cholesterol (*p* < 0.001), low-density lipoprotein (*p* ≤ 0.001), high-density lipoprotein (*p* = 0.011), triglycerides (*p* = 0.035), and free fatty acids (*p* = 0.021). In conclusion, 3-week LCHF-diet reduced fasting glucose, while glucose tolerance was unaffected. A bout of exercise post-intervention did not decrease AUC glucose as it did at baseline. Total cholesterol increased, mainly due to increments in low-density lipoprotein. LCHF-diets should be further evaluated and carefully considered for healthy individuals.

## Introduction

Studies have shown that a low-carbohydrate high-fat (LCHF) diet can be a successful weight-loss tool for overweight and obese individuals (Perez-Guisado et al., 2008; Foster et al., 2010; Bueno et al., 2013; Gu et al., 2013; Bazzano et al., 2014; Moreno et al., 2014) and people with metabolic syndrome or type 2 diabetes (Tay et al., 2015; Steckhan et al., 2016). Studies have also shown that an LCHF diet can improve glycemic control, measured as reduced HbA1c and fasting glucose, and improve blood glucose levels in obese and overweight participants (Tay et al., 2015; Noakes and Windt, 2017; Saslow et al., 2017; Bhanpuri et al., 2018). LCHF diets have also resulted in favorable changes in triglycerides (TG) (Bazzano et al., 2014; Saslow et al., 2017) and high-density lipoprotein (HDL) in obese individuals (Bazzano et al., 2014; Moreno et al., 2014). However, in the aforementioned studies a weight loss of 6–13% occurred, which most likely contributed to the beneficial cardio-metabolic changes in overweight and obese individuals.

There have been concerns that LCHF diets may increase the risk of cardiovascular disease (CVD) due to the increased intake of dietary fats (Mansoor et al., 2016b), as saturated fat has been linked to higher levels of low-density lipoprotein (LDL) with increased vascular dysfunction (Vogel et al., 1997; Mensink et al., 2003; Kim et al., 2005; Grasgruber et al., 2016; Chiu et al., 2017; Lambert et al., 2017; Durrer et al., 2019). Diets high in fat have also been linked to disturbance in gut microbiota with increased gut permeability and inflammation (Murphy et al., 2015; Santos-Marcos et al., 2019).

LCHF diets have been popular among healthy, normal weight people to avoid weight gain (Bugge, 2012; Paoli et al., 2013; Helms et al., 2014; Chang et al., 2017; Zinn et al., 2017), even though knowledge about possible negative health effects in these subjects is scarce. A recent study done on normal-weight type-1 diabetes adults, indicated development of dyslipidemia when adhering to a LCHF diet (Leow et al., 2018). There has been variability in the definition of LCHF diets. A suggest to classification was made in 2013 (Wylie-Rosett et al., 2013), where diets with carbohydrate content of 21–70 g/day is classified as very-low-carbohydrate diet and diets with carbohydrate content of 150–200 g/day is classified as moderately-low-carbohydrate diet.

Previous human studies have shown negative effects of lipid infusion on glucose tolerance (Itani et al., 2002; Hoeg et al., 2011; Hoeks et al., 2012). A higher availability of fatty acids in seen in the blood after an intravenous lipid infusion (≈1.3–2.2 mmol/L), compared to blood levels of fatty acids in studies with LCHF diets without infusion (≈ 0.45 mmol/L) (Numao et al., 2016). A high intake of fat without weight-gain induces insulin resistance in rodents (Jornayvaz et al., 2010; Kinzig et al., 2010), whereas a recent human study showed that a 6 week LCHF diet with 64 percentage of energy (E%) fat, did not decrease insulin sensitivity in slightly overweight males (Lundsgaard et al., 2019).

Exercise has profound effects on metabolism and previous research has shown that an acute bout of endurance exercise results in a transient increase in insulin sensitivity and glucose tolerance (Mikines et al., 1988; Annuzzi et al., 1991; Brestoff et al., 2009; Jensen et al., 2011; Cartee, 2015b; Ortega et al., 2015; Jelstad et al., 2019). Exercise prior to lipid infusion alleviates insulin resistance (Schenk and Horowitz, 2007; Pehmoller et al., 2012; Phielix et al., 2012), but less is known about the effects of exercise on glucose tolerance during an LCHF diet. Aggravation of postprandial glucose metabolism in humans after a high fat diet and exercise needs to be explored.

The main aim of this study was to investigate whether a 3-week LCHF diet with > 75 E% fat, would decrease glucose tolerance and deteriorate the lipid profile in normal weight, young, healthy women. We also hypothesized that a single bout of exercise would counteract possible negative effects of a LCHF diet on glucose tolerance.

## Materials and Methods

### Participants

Twenty-three nutrition students volunteered to participate in a 3-week dietary intervention, and were screened for eligibility. Two were excluded due to impaired glucose tolerance at baseline, and four dropped out during baseline week. Seventeen healthy, moderately trained, normal weight young women were included (Table 1), and gave written informed consent to participate, in accordance with the Declaration of Helsinki. Exclusion criteria were smoking, pregnancy, familial cardio-vascular-disease, diabetes, under- or overweight (BMI < 18.5 or > 25), and high cardiorespiratory fitness (V̇O_2__peak_ > 57 ml/kg/min). This study was approved by the Regional Committee for Medical Research Ethics in Norway (2012/962) and registered in ClinicalTrials.gov, registration number NCT02005224.

**TABLE 1:** Participant characteristics at baseline, after 3 weeks on an LCHF diet and after returning to habitual diet for 1 week.

	**Baseline *n* = 17**	**After 3 weeks of LCHF *n* = 17**	**One week post intervention *n* = 17**
Age (years)	23.5 ± 0.5		
Height (cm)	168 (156–178)		
Weight (kg)	58.8 ± 1.4	56.9 ± 1.3^∗^	58.5 ± 1.3^§^
BMI (kg × m^2^)	21.0 ± 0.4	20.3 ± 0.4^∗^	20.9 ± 0.4^§^
Fat mass (kg)	12.8 (6.6–22.2)	13.2 (6.3–22.6)	12.6 (6.7–21.5)^*§^
Fat%	21.6 ± 1.4	22.3 ± 1.4	21.1 ± 1.3^§^
Fat free mass (kg)	45.2 ± 1.0	44.1 ± 0.9^∗^	46.0 ± 1.0^*§^
TBW (kg)	33.4 ± 0.8	32.0 ± 0.7^∗^	33.1 ± 0.7^§^
V̇O_2peak_ (L/min)	2753 (1732-3161)	2693 (1852–3113)	
V̇O_2peak_ (mL/kg/min)	43.7 ± 1.3	44.6 ± 1.3	
HR_peak_ (beats/min)	182 ± 2	184 ± 2	
FMD (%)	10.7 ± 1.1	13.3 ± 1.0	

*Parametric data are shown as mean ± SEM. Non-parametric data are shown as median (range). LCHF, low-carb-high-fat; BMI, body mass index; BIA, bioelectrical impedance analyses; TBW, total body water; V̇O_2peak_, highest value of oxygen uptake; HR_*peak*_, highest value of heart rate registered; FMD, flow-mediated dilation. ^∗^*P* < 0.05 vs. baseline. ^§^ P < 0.05 vs. LCHF.*

### Design

**FIGURE 1:**
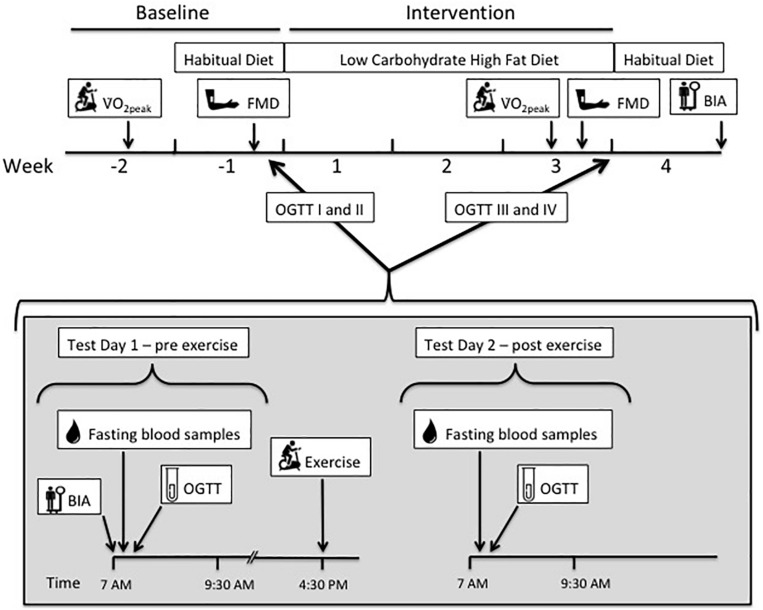
The study timeline illustrates a 2-week baseline period, before 3-week low-carbohydrate high-fat (LCHF) dietary intervention, and 1-week- post intervention with habitual diet. During baseline and intervention, a set of two oral glucose tolerance tests (OGTT) and blood sampling were done on consecutive days, before (test day 1) and after (test day 2) one bout of endurance exercise. Peak oxygen uptake (V̇O_2peak_) and flow-mediated dilatation (FMD) were measured before and after the LCHF diet. Bioelectrical impedance (BIA) measurements were done during each of the diet periods.

The study was a 3-week dietary intervention, performed between September 18, 2012 and November 16, 2012. Baseline data were collected during a 2-week run-in before initiating the LCHF diet intervention. The tests included peak oxygen uptake (V̇O_2__peak_), peak heart rate (HR_peak_) bioelectric impedance analysis (BIA), flow-mediated dilatation (FMD), fasting blood samples and oral glucose tolerance test (OGTT) (Figure 1). Participants also registered their habitual diet during baseline. Post-intervention data were collected at the end of the intervention, followed by one final BIA measurement 1 week after completion of the intervention when participants had returned to their habitual diet. Participants were grouped according to their menstrual cycle and fasting blood sampling and glucose tolerance tests were performed at approximately the same day in luteal phase. Four of the participants used combined oral contraceptive pills, where the remaining 13 participants did not use contraceptive pills. After a pre-exercise oral glucose tolerance test (OGTT I at baseline and III post-intervention), participants performed a bout of exercise in the afternoon, before returning 12 h later for a second, post-exercise OGTT (II at baseline and IV post-intervention).

### V̇O_2__peak_ and HR_peak_

Testing of peak oxygen uptake (V̇O_2__peak_) was done during baseline and post-intervention (Figure 1). The test was completed on an ergometer bicycle (Excalibur Sport Cycle Ergometer, Lode, Netherlands) using an automatic O_2_/CO_2_ analyzer (Moxus Modular Metabolic System, AEI Technologies, Inc.). The V̇O_2__peak_ test started at 50 W with an increase of 15 W every 30 s until exhaustion. Oxygen uptake was measured breath-by-breath, and average calculated in 30-s intervals throughout the protocol. An increase of less than 1 ml/kg/min in V̇O_2_ after two increments in workload, combined with a respiratory exchange ratio (RER) > 1.10 was used as the criterion for V̇O_2__peak_. A second V̇O_2__peak_ test was performed after 16 days on the LCHF diet (Figure 1). The highest heart rate recorded during the V̇O_2__peak_ test was noted as HR_peak_.

### Flow-Mediated Dilation

Endothelial function was assessed by the FMD method (Corretti et al., 2002), before and during the LCHF intervention (Figure 1). The measurements were performed in fasting participants, in a supine position, using a commercially available ultrasound scanner (GE Vingmed Ultrasound, Vivid E9 scanner Horten, Norway). A 10 MHz linear transducer was used for all recordings. Results were automatically analyzed offline from the video files using Brachial Analyzer Software (Medical Imaging Applications LLC, US). In accordance with current guidelines, the transducer was placed in a fixed position, and stabilized with a custom made tripod to ensure recordings were as stable as possible, showing the brachial artery during the recordings. FMD% was calculated as the percentage change between the baseline diameter and the largest diameter of the brachial artery within the first 3 min after 5 min occlusion of the right forearm. All recordings were performed and analyzed by the same experienced ultrasonographer (JH) in accordance with international guidelines (Thijssen et al., 2011).

### Weight, Height, and Body Composition

Weight was measured using BIA (MC 180 MA Multi Frequency, Tanita, Tokyo, Japan) to the nearest 0.1 kg. Height was measured by using a wall mounted stadiometer and registered to the nearest mm. Body composition was estimated using BIA (MC 180 MA Multi Frequency, Tanita, Tokyo, Japan). All measurements were done prior to, and at the end of the LCHF intervention (Figure 1). Weight and body composition were also measured 7 days after completing the dietary intervention, after participants had returned to their habitual diet.

### Fasting Blood Samples – Glucose, Insulin and Lipids

Participants arrived at 06:00 at the laboratory, after a 12-h fast. An intravenous catheter was inserted in the antecubital vein and fasting blood samples were collected (0 min). The samples were collected in serum separator tubes and coagulated for 30 min at room temperature before centrifugation (Eppendorf 5072R, Hamburg, Germany). Samples were stored at 4°C for approximately 4 h until they were analyzed. Glucose, insulin, LDL, HDL and TG were analyzed in serum at Fürst Laboratory, Oslo, Norway (Advia Centaur XPT, Siemens Medical Solutions Diagnostics, Tokyo, Japan).

### D-3-Hydroxybutyrate and Soluble Urokinase-Type Plasminogen Activator Receptor

Samples for free fatty acids (FFA) and D-3-hydroxybutyrate (3-OHB) were collected at 0 (fasting) and 120 min, whereas samples for soluble urokinase-type plasminogen activator receptor (suPAR) were collected at 0 min (fasting). The samples were collected in EDTA tubes and kept on ice before centrifuging at 2500 rpm for 10 min at 4°C. Plasma was pipetted into 1.7 ml microcentrifuge tubes (Croning^®^ Costar^®^) and kept frozen at −80°C until further analysis was conducted.

3-OHB measures were conducted according to the manufacturer’s instructions. Analyses of plasma concentration of 3-OHB were performed at the Department of Clinical Medicine, Diabetes and Hormone Diseases - Medical Research Laboratory, Aarhus University, Denmark, using a kinetic enzymatic method, based on the oxidation of 3-OHB to acetoacetate by the enzyme 3-hydroxybutyrate dehydrogenase (Randox Laboratories Ltd., Crumlin, United Kingdom). Concomitant with this oxidation the cofactor NAD^+^ is reduced to NADH and the associated change in absorbance can be directly correlated with the 3-OHB concentration, using the Cobas c111 system (Roche Diagnostics International Ltd., Rotkreuz, Switzerland).

The content of FFA was measured using an *in vitro* enzymatic colorimetric assay for the quantitative determination of non-esterified fatty acids (NEFA-HR) (Wako Chemicals GmbH, Neuss, Germany) using a Cobas C-111 autoanalyzer (Roche, Germany).

suPAR was measured using a commercially available, enzyme-linked immunosorbent assay kit (ELISA, suPARnostic^®^, Virogates, Copenhagen, Denmark). For analyses, pre-coated immunoassay plates were used, with a monoclonal capture antibody specific to the suPAR component of the sample. A horseradish peroxidase conjugated monoclonal detection antibody (225 μl) that was pre-diluted 1:200 with sample dilution buffer was added to 25 μl plasma and mixed. From this, 100 μl was transferred (in duplicate) to the immunoassay plate and incubated for 1 h. After plate washing, 100 μl of the substrate 3,3′, 5,5′ tetramethylbenzidine was added. After 20 min the reaction was stopped with 100 μl 0.45 M H_2_SO_4_. All incubations were performed at room temperature in the dark. Absorbance was measured spectrophotometrically at 450 nm. Samples were randomly distributed between two kits and were measured in duplicate. All samples had duplicate CVs < 10%.

### Oral Glucose Tolerance Test

After fasting samples were collected, participants ingested 75 g glucose dissolved in 300 ml water over a 5-min timeframe, followed by blood samples at 15, 30, 45, 60, 90, and 120 min. The catheter was kept patent by flushing with 0.9% saline solution after each blood sample collection. OGTTs were performed on two consecutive days; test day one and test day two (Figure 1). Participants refrained from exercise 48 h prior to OGTT on test day one, both at baseline and after the intervention (OGTT I and III).

### HOMA2-Insulin Resistance and Matsuda Index

Glucose and insulin results were used to calculate HOMA2-IR and the Matsuda index as indicators of insulin resistance. HOMA2-IR uses fasting glucose and fasting insulin (Wallace et al., 2004), whereas the Matsuda index uses multiple samples, from 0, 30, 60, 90, and 120 min (Matsuda and Defronzo, 1999; DeFronzo and Matsuda, 2010). Insulin resistance cut-off values for HOMA2-IR and Matsuda were set to 1.2 and 5 respectively (Radikova et al., 2006; Szosland and Lewinski, 2016).

### Exercise

Participants attended an indoor-bicycle exercise session in the afternoon (16.30–17.30) on test day one, after the first OGTT (I at baseline and III post intervention) (Figure 1). The exercise consisted of a 10-min warm-up, followed by 60 min at 75–80% of HR_peak_. Heart rate during the session was recorded using Polar heart rate monitors (RA800CX, Polar Electro Oy, Finland). A qualified instructor supervised the exercise.

### Dietary Intervention and Monitoring

Daily energy requirements were calculated using basal metabolic rate (BMR) estimated with BIA (MC 180 MA Multi Frequency, Tanita, Tokyo, Japan) (Verney et al., 2015) multiplied by the coefficient of activity calculated according to the daily physical activity level (PAL) of each participant (Ategbo et al., 2005). Participants registered their habitual diet for 1 week during baseline (Figure 1). Registration of the habitual diet showed that participants had a normal prudent diet, recommended by the Norwegian Health Authorities. The diet registration during baseline also served as a control between actual energy intake and calculated requirement. Food was weighed on an electronic scale (1 g precision), dietary intake was registered in an online dietary registration program^1^ (Somebody AS © 2008–2016), and total energy intake, carbohydrate, fat and protein intake were calculated. For calculating the percentage of mono-, poly- and saturated fats, the dietary registration program “Mat På Data” (version 5.1; The Norwegian Food Safety Authority 2009) was used.

During the intervention, participants consumed an energy-balanced, LCHF diet. Participants were instructed to ingest > 75 E% fat, ≤20 E% protein and carbohydrates ≤5 E%. Participants received LCHF recipes before the intervention and were supplied with full-fat dairy products, vegetables, low-carb bread-mix. Participants had an option of using fat emulsion formula (Fresubin^®^ 5 kcal SHOT, Fresenius Kabi, Bad Homburg, Germany). Preparation of food was done by the participants. Physical activity, food and energy intake registered and were monitored by research leader every evening during the LCHF intervention, and adjusted if needed according to energy requirements. This was important as the energy-balanced diet was designed to keep participants’ weight stable through the intervention.

Participants consumed a standardized meal after the bout of exercise on test day 1 prior to the diet intervention, consisting of 16 E% fat, 27 E% protein and 57 E% carbohydrate. After the 3-week dietary intervention, participants consumed a standardized fat-rich diet after the exercise on test day 1, consisting of 75 E% fat, 24 E% protein and 1 E% carbohydrate. All meals were energy-balanced according to individual requirements.

### Ketosis

Compliance to the LCHF diet was monitored by measuring ketone bodies in urine with urine sticks (Ketostix 2880, Bayer, Berlin, Germany) every morning from day one of the LCHF diet. Participants compared the strip to a color chart, on which the scale was trace (0.5 mmol/L), small (1.5 mmol/L), moderate (4 mmol/L) and large (8–16 mmol/L) and registered the results in the online dietary registration program.

### Statistical Analyses

Statistical analyses were performed using SigmaPlot for Windows software (version 12.5; Systat Software Inc.). The effects of diet were evaluated using repeated-measures analysis of variance (ANOVA). To examine the main effects of treatment and/or time, a two-way ANOVA was used. One-way ANOVA was used to analyze change in weight and body composition (baseline, LCHF, 1 week post intervention). Otherwise a two-tailed, paired *t*-test was used for parametric data and the Mann–Whitney test was applied for non-parametric data. Shapiro–Wilk was used for testing normal distribution of data. Significance was set at *p* ≤ 0.05. Values are presented as mean values ± SEM, unless otherwise specified. AUC was calculated using the trapezoidal rule (timepoints 0, 15, 30, 45, 60, 90, 120 min).

## Results

Of the 23 participants screened at baseline, 17 completed the study (74%). These were healthy, moderately trained normal weight young women (58.8 ± 1.4 kg) aged 23.5 ± 0.5 years (Table 1). Despite the fact that the diet during the intervention was energy balanced in accordance to predicted daily metabolic demand (BMR^∗^PAL), body weight was reduced from 58.8 ± 1.4 to 56.9 ± 1.3 kg (*p* < 0.001) after the intervention. According to BIA measures the body weight reduction was mainly due to loss in total body water (33.4 ± 0.8 kg to 32.0 ± 0.7 kg, *p* < 0.001), and fat-free mass (45.2 ± 1 kg to 44.1 ± 0.9 kg, *p* = 0.003), with no change in % body fat or total fat mass. BMI decreased from 21.0 ± 0.4 to 20.3 ± 0.4 kg/m^2^ (*p* = ≤ 0.001) following the LCHF diet. Body weight and body composition were back to baseline values 7 days after participants had returned to their habitual diets.

### Diet Records

**FIGURE 2:**
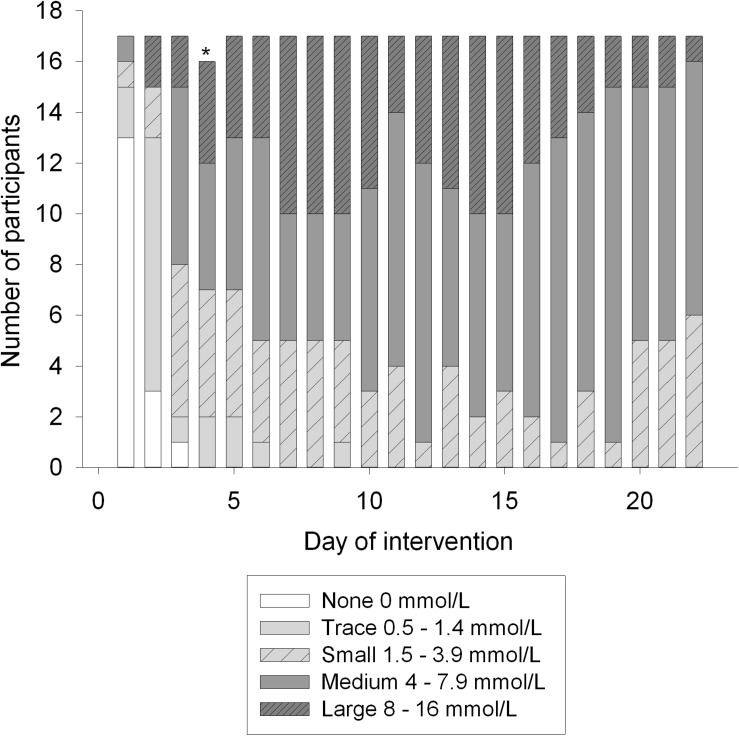
Ketone bodies in urine during the intervention, measured with semi-quantitative method (Ketostix). Amount of ketones evaluated by strip-color divides participants into five groups; none, trace, small, medium and large. ^∗^Indicates a missing value.

Prior to the intervention, E% carbohydrate was 45 ± 2. The E% carbohydrate was reduced to 3 ± 0 (*p* < 0.001) after the LCHF diet (Table 2). On the contrary, E% fat was increased from 35 ± 2 to 78 ± 1 (*p* < 0.001) with increased E% intake of both saturated and unsaturated fats. E% saturated fat increased from 10 ± 1 to 30 ± 1 (*p* < 0.001), whereas E% mono- and polyunsaturated fats increased from 9 ± 1 to 28 ± 1 (*p* < 0.001) and 6 ± 1 to 11 ± 1 (*p* < 0.001), respectively. No changes were seen in E% protein intake or in total energy intake. Energy intake/energy requirements showed that all participants were in energy balance during the diet intervention. Ketosis was observed from day 3 to 22, indicated by urine strips (Figure 2).

**TABLE 2:** Daily energy intake, energy expenditure and dietary composition during baseline and the 3-week LCHF diet.

	**Baseline (1 week) *n* = 17**	**LCHF (3 weeks) *n* = 17**
Energy intake (kcal/24 h)	2137 ± 48	2186 ± 44
Energy expenditure (kcal/24 h)	2177 ± 59	2201 ± 63
Energy balance (kcal/24 h)	−40 ± 27	−16 ± 47
E% Protein	20 ± 1	19 ± 1
E% Carbohydrate	45 ± 2	3 ± 0^∗^
E% Added sugars	5 ± 0	0 ± 0^∗^
E% Fat	35 ± 2	78 ± 1^∗^
E% Saturated fats	10 ± 1	30 ± 1^∗^
E% Monounsaturated fats	9 ± 1	28 ± 1^∗^
E% Polyunsaturated fats	6 ± 1	11 ± 1^∗^

*Data are shown as mean ± SEM. Dietary compositions were calculated during weeks 1 and 3, respectively. E%, percentage of total energy intake. ^∗^*P* ≤ 0.05 vs. baseline.*

### Effect of LCHF Diet Intervention (OGTT I vs. III)

#### Glucose Tolerance

**FIGURE 3:**
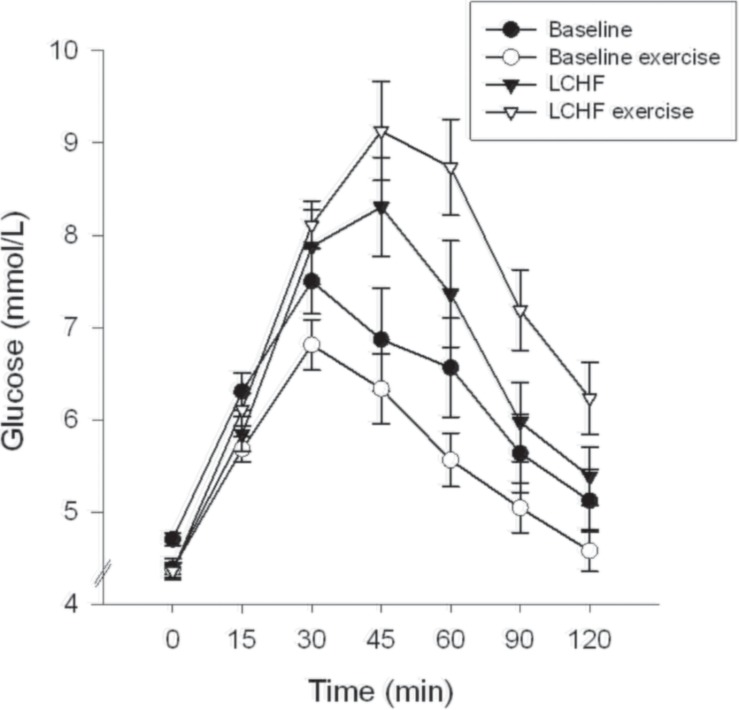
Glucose time course. The curves show glucose levels at 0, 15, 30, 45, 60, 90, and 120 min. Oral glucose tolerance tests (OGTT) were performed at baseline, and after the low-carbohydrate high-fat (LCHF) diet intervention. A single bout of endurance exercise at 75-80% of peak heart rate (HR_peak_) was done in the afternoon on test day 1 in both periods.

**FIGURE 4:**
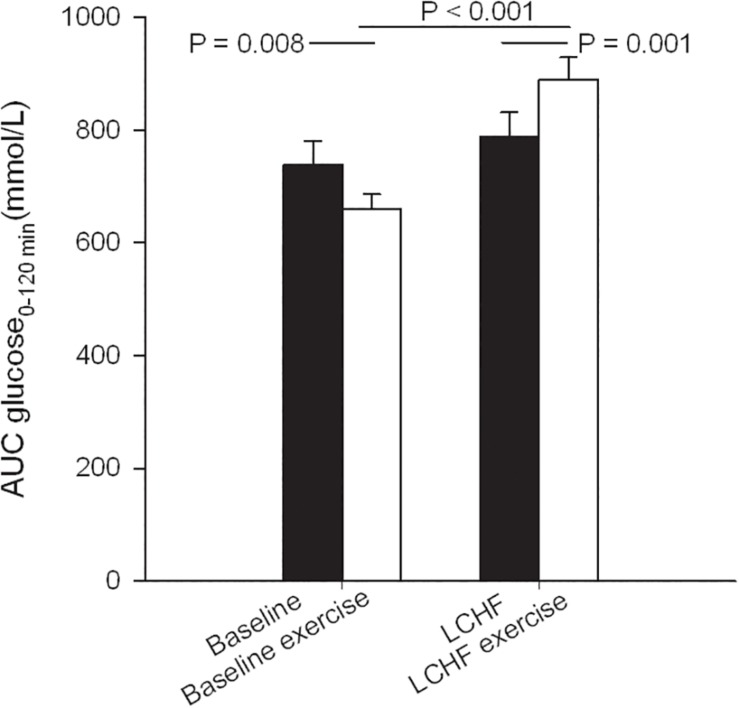
Glucose area under the curve (AUC) during oral glucose tolerance tests (OGTT) that were performed at baseline, and after the low-carbohydrate high-fat (LCHF) intervention. A single bout of endurance exercise at 75–80% of peak heart rate (HR_peak_) was done in the afternoon on test day 1 in both periods.

**FIGURE 5:**
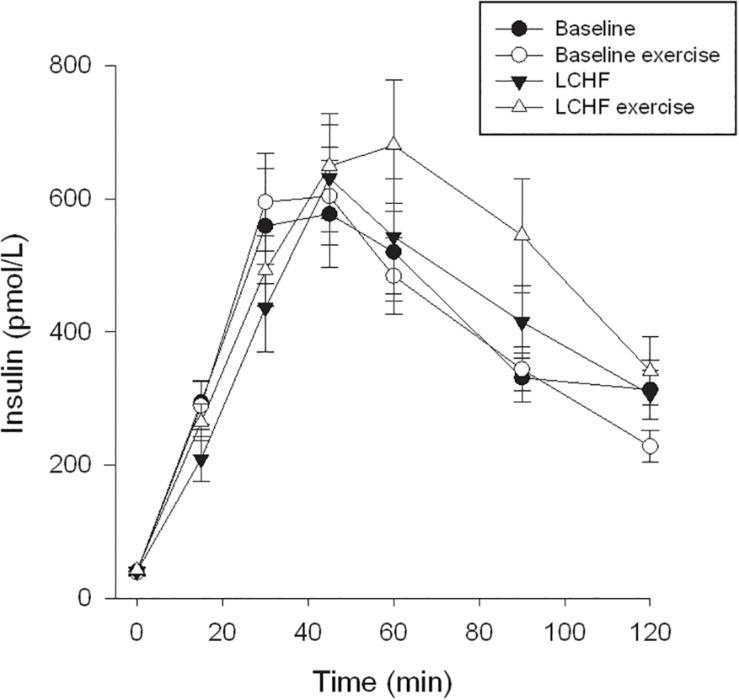
Insulin time course. The curves show insulin levels at 0, 15, 30, 45, 60, 90, and 120 min. Oral glucose tolerance tests (OGTT) were performed at baseline, and after the low-carbohydrate high-fat (LCHF) intervention. A single bout of endurance exercise at 75–80% of peak heart rate (HR_peak_) was done in the afternoon on test day 1 in both periods.

**FIGURE 6:**
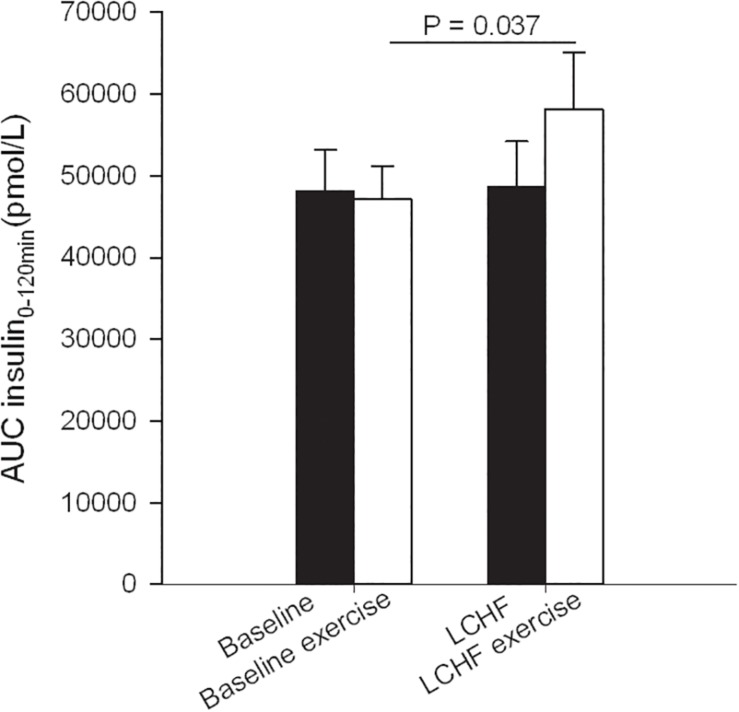
Insulin area under the curve (AUC) during oral glucose tolerance tests (OGTT) that were performed at baseline, and after the low-carbohydrate high-fat (LCHF) intervention. A single bout of endurance exercise at 75–80% of peak heart rate (HR_peak_) was done in the afternoon on test day 1 in both periods.

Fasting glucose at baseline was 4.7 ± 0.1 mmol/L and decreased to 4.4 ± 0.1 mmol/L after 3 weeks on LCHF diet (*p* ≤ 0.001) (Table 3). The 120-min glucose level during the OGTT did not change comparing baseline and post intervention (Figure 3 and Table 3). AUC glucose was unaffected after the diet (Figure 4). Further on, the diet did not cause changes in fasting insulin (Table 3), insulin at 120 min (Figure 5 and Table 3), or AUC insulin (Figure 6). HOMA2-IR and Matsuda index were unchanged after the dietary intervention (Table 3).

**TABLE 3:** Fasting and 120-min values for glucose and insulin during an oral glucose tolerance test.

	**Baseline *n* = 17**	**Baseline after Exercise *n* = 17**	**LCHF *n* = 17**	**LCHF after Exercise *n* = 17**
**Glucose (mmol/L)**				
Fasting (0 min)	4.7 ± 0.1	4.4 ± 0.1^∗^	4.4 ± 0.1^∗^	4.4 ± 0.1
120 min	5.1 ± 0.3	4.6 ± 0.2^∗^	5.4 ± 0.3	6.2 ± 0.4^**§^
**Insulin (pmol/L)**				
Fasting (0 min)	40 ± 5	39 ± 6	40 ± 6	39 ± 6
120 min	313 ± 45	223 ± 25	317 ± 37	352 ± 57^∗∗^
**Matsuda index**	7.6 ± 1.1	9.9 ± 2.3	7.5 ± 0.9	6.0 ± 0.6^∗∗^
**HOMA2-IR**	0.74 ± 0.08	0.71 ± 0.11	0.73 ± 0.11	0.76 ± 0.11

*Insulin sensitivity was calculated as Matsuda and HOMA2- IR. Values shown for baseline and after 3 weeks on a LCHF diet, before and after one bout of exercise. Data are shown as mean ± SEM. ^∗^*P* ≤ 0.05 vs. Baseline, ^∗∗^*P* ≤ 0.05 vs. baseline after exercise, ^§^*P* ≤ 0.05 vs. LCHF.*

#### FFA and 3-OHB

FFA fasting levels increased from 0.54 ± 0.04 to 0.80 ± 0.07 mmol/L (*p* = 0.02), comparing baseline values with post-intervention values (Table 4). At 120 min during the OGTT, FFA had increased from 0.06 ± 0.00 to 0.33 ± 0.06 mmol/L (*p* < 0.001) comparing baseline values with post-LCHF. Δ FFA during the OGTT was similar at baseline and after the LCHF intervention. An increase was seen in fasting 3-OHB, from 0.13 ± 0.03 to 1.14 ± 0.19 mmol/L (*p* < 0.001) comparing baseline values to post-LCHF (Table 4). At 120 min 3-OHB was increased from 0.02 ± 0.00 to 0.24 ± 0.07 mmol/L (*p* < 0.001) as a result of the intervention, and the Δ 3-OHB was larger after the intervention as the values went from 0.12 ± 0.03 to 0.90 ± 0.18 mmol/L (*p* < 0.001) (Table 4).

**TABLE 4:** FFA and 3-OHB, fasting (0 min) and 120 min values during an oral glucose tolerance test (measured in plasma samples).

	**Baseline *n* = 17**	**Baseline after Exercise *n* = 17**	**LCHF *n* = 17**	**LCHF after Exercise *n* = 17**
**FFA (mmol/L)**				
Fasting (0 min)	0.54 ± 0.04	0.55 ± 0.06	0.80 ± 0.07^∗^	0.86 ± 0.08^∗∗^
120 min	0.06 ± 0.00	0.06 ± 0.00	0.33 ± 0.06^∗^	0.23 ± 0.04^**§^
Δ (120 – 0 min)	0.48 ± 0.04	0.50 ± 0.06	0.47 ± 0.11	0.63 ± 0.08
**3-OHB (mmol/L)**				
Fasting (0 min)	0.13 ± 0.03	0.12 ± 0.02	1.14 ± 0.19^∗^	1.17 ± 0.14^∗∗^
120 min	0.02 ± 0.00	0.02 ± 0.00	0.24 ± 0.07^∗^	0.15 ± 0.03^**§^
Δ(120 – 0 min)	0.12 ± 0.03	0.11 ± 0.02	0.90 ± 0.18^∗^	1.02 ± 0.12^∗∗^

*Values shown for baseline and after 3 weeks on a LCHF diet, before and after one bout of exercise. Data are shown as mean ± SEM. FFA, free fatty acids; 3-OHB, D-3-hydoxybutyrate; Δ, difference between 120 and 0 min. ^∗^*P* ≤ 0.05 vs. Baseline, ^∗∗^*P* ≤ 0.05 vs. baseline after exercise, ^§^*P* ≤ 0.05 vs. LCHF.*

### Lipids, suPAR and FMD

Prior to the intervention, all participants had cholesterol levels within the normal range (Table 5). The intervention resulted in an increase in fasting serum concentrations of total cholesterol from 4.4 ± 0.2 up to 5.6 ± 1.7 mmol/L (*p* < 0.001). TG increased after the intervention, from 0.68 ± 0.06 to 0.82 ± 0.05 mmol/L (*p* = 0.035). HDL and LDL increased in response to the LCHF diet, from 1.8 ± 0.1 and 2.3 ± 0.1 mmol/L, to 2.0 ± 0.1 and 3.2 ± 1.2 mmol/L respectively (*p* = 0.011 and <0.001). The LDL/HDL ratio was increased from 1.36 ± 0.11 to 1.64 ± 0.13 mmol/L (*p* = 0.043) comparing baseline with post-intervention values. The suPAR concentration decreased by 6.7% in response to the intervention, from 2.38 ± 0.06 to 2.22 ± 0.07 ng/mL (*p* = 0.018) (Table 5), whereas there was no change in FMD (Table 2).

**TABLE 5:** Fasting blood parameters, at baseline and after 3 weeks on a LCHF diet.

	**Baseline *n* = 17**	**LCHF *n* = 17**
Total cholesterol (mmol/L)	04.4 ± 0.2	5.6 ± 1.7^∗^
LDL (mmol/L)	2.3 ± 0.1	3.2 ± 1.2^∗^
HDL (mmol/L)	1.8 ± 0.1	2.0 ± 0.1^∗^
TG (mmol/L)	0.68 ± 0.06	0.82 ± 0.05^∗^
LDL/HDL (mmol/L)	1.36 ± 0.11	1.64 ± 0.13^∗^
suPAR (ng/mL)	2.38 ± 0.06	2.22 ± 0.07^∗^

*Data are shown as mean ± SEM. Baseline; baseline values, LCHF, low-carbohydrate high-fat diet. LDL, low density lipoprotein; HDL, high density lipoprotein; TG, triglycerides; suPAR, soluble urokinase-type plasminogen activator receptor. ^∗^*p* = ≤ 0.05 vs. baseline.*

### V̇O_2__peak_, HR_peak_ and RER

**FIGURE 7:**
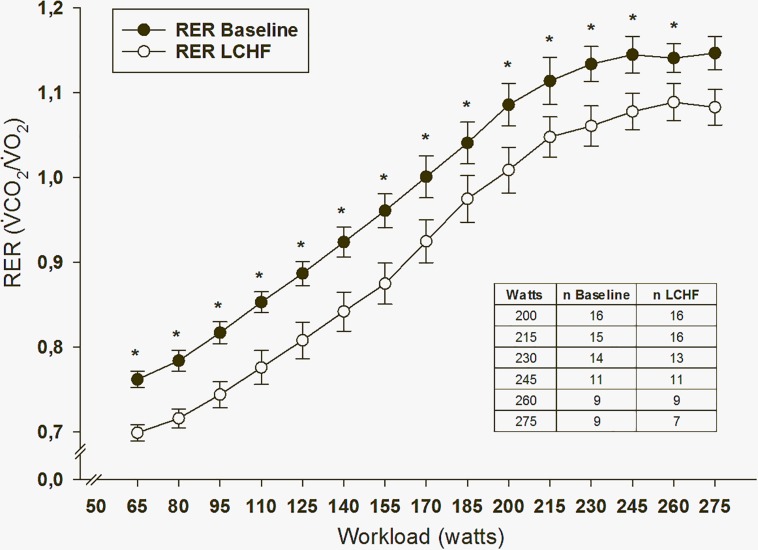
Respiratory exchange ratio (RER) values during peak oxygen uptake test (V̇O_2peak_). ^∗^*p* = ≤ 0.05 baseline vs. low-carbohydrate high-fat (LCHF) intervention. The number of participants drops when the workload increases due to individual difference in physical fitness.

No changes were seen in V̇O_2__peak_ or HR_peak_ when comparing baseline with LCHF values (Table 2). Prior to the intervention, participants reached an RER value of 1 at a workload of around 170 watts, whereas after the intervention, the same workload resulted in a lower RER value (*p* < 0.001) (Figure 7).

### Effect of Exercise Prior to the Diet Intervention (OGTT I vs. II)

#### Glucose Tolerance

After a bout of exercise prior to the intervention, fasting glucose decreased from 4.7 ± 0.1 mmol/L to 4.4 ± 0.1 mmol/L (*p* < 0.001) (Table 3). Glucose at 120 min during the OGTT was lower after a bout of exercise, compared to baseline values the previous day, as it went from 5.1 ± 0.3 to 4.6 ± 0.2 mmol/L (*p* = 0.031). AUC glucose decreased from 739 ± 41 to 661 ± 25 (mmol/L∙120 min^–1^) (*p* = 0.008) prior to the intervention, comparing the pre-exercise day with the post-exercise day (Figure 4). Indexes for insulin sensitivity, measured as HOMA2-IR and Matsuda, were unaffected after a bout of exercise prior to the dietary intervention (Table 3).

#### FFA and 3-OHB

No changes were seen in FFA or 3-OHB comparing baseline and post-exercise values prior to the intervention.

### Effect of Exercise After the Diet Intervention (OGTT III vs. IV)

#### Glucose Tolerance

After a 3-week LCHF dietary intervention, no changes were seen in fasting glucose after a bout of exercise (Table 3). On the other hand, glucose levels at 120 min were increased from 5.4 ± 0.3 to 6.2 ± 0.4 mmol/L (*p* = 0.001) comparing pre-exercise day with post-exercise day after the LCHF intervention (OGTT III vs. OGTT IV). AUC glucose increased from 789 ± 43 to 889 ± 40 mmol/L∙120min^–1^ (*p* = 0.001) comparing the pre-exercise OGTT (III) with the post-exercise OGTT (IV) after the diet intervention (Figure 4). Fasting insulin, insulin levels at 120 min and AUC insulin were all unaffected after a bout of exercise after the diet intervention (Table 3 and Figure 6). No changes were seen in HOMA2-IR or the Matsuda index (Table 3).

#### FFA and 3-OHB

Fasting FFA levels did not change comparing pre-exercise and post-exercise days after the intervention (Table 4). At 120 min during the OGTT post-exercise, FFA decreased from 0.33 ± 0.06 to 0.23 ± 0.04 mmol/L (*p* = 0.021), whereas Δ FFA was unaffected. A similar pattern was seen in 3-OHB concentrations where 120-min 3-OHB levels during OGTT decreased from 0.24 ± 0.07 to 0.15 ± 0.03 mmol/L (*p* = 0.023) (Table 4), but fasting level did not change. Δ 3-OHB was unaffected when comparing pre-exercise with post-exercise day, after the intervention.

### Effect of Diet Intervention and Exercise (OGTT II and IV)

#### Glucose Tolerance

When comparing post-exercise days, prior to and after the intervention, no changes were seen in fasting glucose (Table 3). There was an increase in glucose level at 120 min, from 4.6 ± 0.2 to 6.2 ± 0.4 mmol/L (*p* < 0.001) and an increase in AUC glucose from 661 ± 25 to 889 ± 40 mmol/L∙120min^–1^ (*p* < 0.001) comparing post-exercise days prior to, and after the intervention (Figure 4). No changes were seen in fasting insulin, but an increase was observed in insulin level at 120 min, from 223 ± 25 to 352 ± 57 pmol/L (*p* = 0.018) (Table 3). AUC insulin increased from 47199 ± 3954 to 58177 ± 6889 pmol/L∙120min^–1^ (*p* = 0.037) comparing post-exercise days prior to and after the intervention (Figure 6). A decrease was observed in Matsuda index from 9.9 ± 2.3 to 6.0 ± 0.6 (*p* = 0.003) (Table 3), whereas HOMA-IR was unaffected.

#### FFA and 3-OHB

Fasting FFA increased comparing the post-exercise day at baseline with the post-exercise day after the intervention, where values changed from 0.55 ± 0.06 to 0.86 ± 0.08 mmol/L (*p* = 0.011) (Table 4). FFA at 120 min during the OGTT increased from 0.06 ± 0.00 to 0.23 ± 0.04 mmol/L (*p* = 0.005), where Δ FFA did not change. Fasting 3-OHB levels increased from 0.12 ± 0.02 to 1.17 ± 0.14 mmol/L (*p* < 0.001) comparing post-exercise day before and after the intervention (Table 4). At 120 min during the OGTT, 3-OHB levels increased; from 0.02 ± 0.00 to 0.15 ± 0.03 mmol/L (*p* = 0.020), and Δ 3-OHB increased, from 0.11 ± 0.02 to 1.02 ± 0.12 mmol/L (*p* < 0.001) as a result of the intervention, comparing post-exercise days.

## Discussion

The primary aim of the present study was to investigate whether a 3-week LCHF diet with >75 E% fat, would decrease glucose tolerance and negatively affect the lipid profile in normal weight, young, healthy women. The second aim of the study was to investigate if a single bout of exercise would counteract possible negative effects of an LCHF diet on glucose tolerance.

### Diet

In the present study normal weight, healthy, young women ingested energy-balanced LCHF diets for 3 weeks. Participants reported daily food and beverage intake, and all physical exercise. This process was undertaken to monitor energy intakes and energy requirements, and adjustments were made when necessary to keep the diet energy-balanced. Diet records from the LCHF period showed high intakes of total fat (78 E%) and low intake of carbohydrates (3 E%) compared to baseline values. Protein intake remained the same prior to and during the intervention (19 E%). Previous LCHF studies have usually allowed and included an increase in protein intake (Volek et al., 2004; Brinkworth et al., 2009a; Foster et al., 2010; Naude et al., 2014). Urine ketone bodies indicated good adherence to the LCHF diet. Ketone bodies were elevated from day three and throughout the intervention. This semi-quantitative test gives a good indication of the level of ketone bodies and urine measurements are relatively reliable for such purposes as ketogenic diet studies. The subjective evaluation of strip-color compared to scale-color can cause a bias in results, but urine measurements were done to indicate diet adherence, rather than to quantify precise values (Urbain and Bertz, 2016).

### Body Composition

Although careful control of food intake and exercise was conducted to ensure energy balance, participants did lose on average ∼ 2 kg, where fat mass was unchanged (+400 g) but total body water (−1400 g) and fat-free mass (−1100 g) were reduced. The reduction in total body water and the slight increment in total fat mass could be explained by loss of water due to reductions in glycogen stores. Studies on LCHF diets have shown that resting muscle glycogen stores are reduced to ∼45% of the values seen on a high-carbohydrate diet (Burke, 2015), where 1 g of glycogen binds 3 g of water (Fernandez-Elias et al., 2015). Muscle biopsies for measurement of muscle-glycogen were not collected in this study, but similar drops in total body water and fat-free mass have previously been seen in participants on LCHF diets (Yancy et al., 2004). It is important to consider whether the method of body composition assessment could affect the changes seen after LCHF diets, as both DXA and BIA measure fat-free mass, which can be affected by water distribution (Tinsley and Willoughby, 2016). Body weight, fat-free mass and total body water returned to baseline values 1-week post intervention, when participants were back on their habitual diets. The results support the hypothesis that the changes in fat-free mass were caused by a transient drop in total body water.

### Fasting Glucose, Insulin, FFA and 3-OHB

The LCHF diet decreased fasting glucose levels, but did not change fasting insulin in normal weight healthy young females. Previous LCHF studies have shown greater clinical benefits than low-fat diets among overweight and obese participants (Nielsen and Joensson, 2006; Brinkworth et al., 2009b), but it has been debated whether such changes are consequences of the diet, or whether it is simply the weight reduction (Stevens Ohlson, 2006). Reduced intake of carbohydrates requires less insulin secretion, thereby lowering both glucose and insulin in the blood. Thus, the dysregulated state of glucose and insulin often seen in obesity can be corrected with both hypocaloric diets and LCHF diets (Mansoor et al., 2016a). LCHF diets have been superior to low-fat diets in terms of reductions in fasting glucose (Noakes and Windt, 2017), probably due to both weight reduction and reduced carbohydrates in the diet. The daily carbohydrate intake in our study was 3 E%, which is about 15 g. Reduced fasting glucose, together with unaffected fasting insulin after the intervention, most likely reflects an extremely low carbohydrate intake, and a reduced requirement for insulin to stimulate glucose uptake.

The LCHF diet resulted in higher FFA levels when compared to baseline values. Elevation of fasting FFAs has been linked to insulin resistance in skeletal muscle in obese patients, as it is thought to cause lipotoxicity with intramyocellular accumulation of TG, and fatty acid metabolites such as diacylglycerol and ceramid (Chow et al., 2010; Samuel et al., 2010). Lipid infusion increases FFA concentration significantly above physiological levels and causes insulin resistance, a model often used in research. Infusion studies show that the relationship between FFA and insulin resistance is dose-dependent, but the reduction in insulin sensitivity is detectable at levels that are well within the physiological range (Chow et al., 2010). The increment in FFA we observed after the intervention in our study, reached levels often seen in patients with T2DM who suffer from insulin resistance (Belfort et al., 2005).

The consequence of a LCHF diet is reduced amounts of glucose, higher availability of FFA and increments in fasting 3-OHB. This effect of LCHF on 3-OHB was observed in the current study, and has previously been reported by other research groups (Fuehrlein et al., 2004; Brinkworth et al., 2009a). Higher levels of 3-OHB after the intervention confirmed a shift in metabolism toward ketosis and fat utilization.

### Fasting Lipids

The LCHF intervention resulted in a 20% increment in TG levels. Previous studies of overweight participants have shown reductions in triglyceride levels on both hypocaloric normal diets and hypocaloric low-carbohydrate diets (Nordmann et al., 2006; Brinkworth et al., 2009a). However, overweight and obese participants usually have distorted lipid profiles at baseline, and hypocaloric diets result in a decrement in TG. The TG elevation we registered can likely be attributed to the nearly doubled intake of saturated fat, the isocaloric diet and the normal weight, healthy participants. The post-intervention levels of TG in our participants were still within normal values and below the primary prevention target for lipid-lowering treatment.

The increment in LDL (39%) has previously been seen in other LCHF studies and can be explained by increased saturated fats in the diet (Mensink et al., 2003; Chiu et al., 2017; Lambert et al., 2017; Noakes and Windt, 2017). However, the effect of LCHF diets on LDL among overweight and obese participants is controversial. Indeed, LCHF diet studies that resulted in weight reduction have not shown any negative effect on LDL (Foster et al., 2010; Bazzano and Hu, 2015; Tay et al., 2015; Saslow et al., 2017). The discrepancy in the effect of LCHF diets on LDL can be explained by differences in baseline values and/or the weight reduction seen after the intervention. The degree of dyslipidaemia varies greatly, but in obese and overweight participants, baseline levels are often well above target levels for primary prevention. The participants in our study had normal LDL levels prior to the intervention. After the intervention, 59% of the participants had higher LDL levels than the target for primary prevention of CVD, which calls for a close attention when it comes to this type of LCHF diet.

In the current study, HDL levels were increased by 11% after the LCHF intervention. A similar increase has been seen in other studies done on normal weight adults (Sharman et al., 2002; Volek et al., 2003; Urbain et al., 2017). Although the modest increase in HDL would have favorable effects on CVD risk, the increment in other lipids could abolish the protective effects of raised levels of HDL.

The larger increment in LDL than HDL in our study resulted in a higher LDL/HDL ratio, shifting toward a more negative profile. Thus, the positive impact on blood lipids due to weight loss previously seen in overweight and obese participants on LCHF diets (Hu et al., 2012; Ajala et al., 2013; Noakes and Windt, 2017) does not apply to our study participants. Healthy, normal weight adults consuming a LCHF diet on a regular basis might be at increased risk of a less favorable lipid profile, according to others and our findings (Urbain et al., 2017). Even slightly elevated LDL cholesterol may have a negative impact on cardiovascular risk (Ference et al., 2017). The increments in LDL and the LDL/HDL ratio should not be ignored, even in healthy young people. The best way to protect against CVD is lifelong low levels of LDL (Linsel-Nitschke et al., 2008; Goldstein and Brown, 2015).

### Fasting FMD and suPAR

Endothelial dysfunction and reduced vasodilatation have been identified as an early feature of atherosclerosis. Both are linked to high levels of LDL (Schwingshackl and Hoffmann, 2013) and hyperglycemia (Kawano et al., 1999). Low-carbohydrate diets have been associated with a significant decrease in FMD when compared to a low-fat diet (Varady et al., 2011). No changes in FMD were seen after the intervention in our young healthy participants. Previous studies among dyslipidemic, overweight and obese individuals have shown a decrease in FMD after a LCHF diet (Schwingshackl and Hoffmann, 2013). Overweight and obese participants often have underlying conditions that have taken years to develop. Our results show that a short-term LCHF diet does not reduce dilatation of the brachial artery in young, normal weight healthy females.

suPAR is a chemotactic molecule that promotes activation of the inflammatory and immune system. It has a prognostic value as a biomarker in different settings, including CVD (Hodges et al., 2015). Serum levels of suPAR in healthy controls have been registered in the range 2.24 – 2.8 ng/mL (Shen et al., 2015; Wu et al., 2015; Vasarhelyi et al., 2016). A Danish general population-based study reported that suPAR levels increase with age (Haupt et al., 2014). Higher suPAR levels were also recorded in participants eating unhealthy diets, with a strong association with higher BMI. The significant reduction we observed after the LCHF intervention was surprising. A shift in n-6/n-3 ratio might influence the serum suPAR concentrations. A high intake of n-6 PUFA, along with low intake of n-3 PUFA, causes a shift in the physiological state to one that is proinflammatory and prothrombotic. This increases vasospasm, vasoconstriction, and blood viscosity and can cause development of diseases associated with these conditions (Patterson et al., 2012). Unfortunately we do not have data on n-6/n-3 ratio in the present study, but the increased intake of polyunsaturated fatty acid was mainly from full fat dairy products and fat emulsion shots, leading to an increased intake of omega 6. An increase in omega 6 should enhance the inflammatory marker suPAR. It is therefore unlikely that a favorable shift in the n-6/n-3 ratio can explain the observed results. The unexpected finding may be a result of the short LCHF intervention. The participants, healthy, normal weight young females had no signs of CVD prior to the study. CVD is a condition that will develop over many years and although suPAR outscores CRP as a biomarker prognosticating CVD, the duration of the dietary intervention may have been too short to stimulate a suPAR response.

### Effect of LCHF Diet

#### Glucose and Insulin

Our results indicate that the overall metabolic regulation of glucose and insulin after one glucose load was not affected by the LCHF diet. Both AUC glucose and AUC insulin were unaffected and all participants reached glucose levels within normal range by the end of the OGTT. Further on, Matsuda index was not affected by the diet intervention. Similar results were seen in a resent study where a high-fat eucaloric feeding did not cause reduction in glucose tolerance in healthy slightly overweight males (Lundsgaard et al., 2019).

#### FFA and 3-OHB

The current study observed the same reduction (absolute values) in FFA during an OGTT at baseline and after the dietary intervention. Previous LCHF studies show conflicting results for FFA suppression during a glucose load. A 72-h LCHF (fat E% 69) showed complete suppression of lipolysis during an OGTT (Numao et al., 2016). Another 69-h LCHF study (fat E% 83) showed a reduced suppression of lipolysis during an intravenous glucose tolerance test (IVGTT) (Johnson et al., 2006) with a similar FFA decrease as in the current study. Fasting induces a similar metabolic shift as seen in LCHF studies; higher lipid oxidation and ketone production. Studies where OGTT was performed after 6 days of fasting have previously been unable to lower plasma FFA to the same absolute levels as seen after an overnight fast (Cahill et al., 1966; Goschke, 1977). Even after meal-induced secretion of insulin after 3 days of fasting, insulin did not suppress FFA to post-prandial levels compared to levels after an overnight fast (Horton and Hill, 2001). The abovementioned results, along with the results from our study, indicate some degree of insulin resistance in adipose tissue, whereas the 3-OHB results suggest unaffected hepatic insulin sensitivity, as 3-OHB levels were nearly down to baseline values after an OGTT after the intervention.

### Effect of Exercise on Glucose Tolerance Prior to the LCHF Intervention

In the present study we found a positive effect of a bout of exercise on fasting glucose and AUC glucose prior to the LCHF intervention (OGTT I vs. OGTT II). The same effect has been confirmed in previous studies (Jensen et al., 2011; Bird and Hawley, 2016). The literature is conflicting regarding the effect of exercise on glucose tolerance; some studies have shown no significant effect of acute exercise on glucose tolerance (Rose et al., 2001; Knudsen et al., 2014; Malin et al., 2016). Indeed, the timing of the OGTT after exercise is crucial. Studies on the immediate effect of exercise show higher rates of oral glucose tolerance, measured as AUC, and studies where OGTTs were run 10–24 h after a bout of exercise show increased glucose tolerance in both overweight and normal weight participants (King et al., 1995; Nelson and Horowitz, 2014; Bird and Hawley, 2016). The positive effect of exercise on fasting glucose and AUC glucose seen 14 h after a bout of exercise was not accompanied by significant changes in fasting insulin, or in AUC insulin. This might be linked to AMPK, which is thought to be important for the ability of exercise to increase GLUT4 translocation and insulin sensitivity in skeletal muscle (Cartee, 2015a; Kjøbsted et al., 2016), as exercise increases glucose disposal in response to physiological insulin concentrations.

### Effect of Exercise on Glucose Tolerance After the LCHF Intervention

Previous studies show conflicting results when it comes to the positive response in glucose tolerance after a bout of exercise, following high-fat feeding. Indeed, studies have shown that high-fat diet attenuated the positive effect of a bout of endurance exercise on glucose tolerance in sedentary, normal weight young men (Skovbro et al., 2011). Other studies have also shown that high fat diets blunt many of the positive effects of exercise on glucose tolerance (Simonsen et al., 1991; Helge et al., 1996; Helge, 2002). In contrast, it has been shown that a single high-fat meal did not prevent the improvement in glucose tolerance after an acute bout of resistance exercise (Shaw et al., 2011). Our data showed a 34% increment in AUC glucose 14 h after a bout of exercise, with no changes in fasting glucose. The results demonstrate that the positive effect of an acute bout of endurance exercise seen prior to the intervention was blunted after a bout of exercise, after the LCHF diet. It is noteworthy that insulin was not affected by a bout of exercise after the intervention. The increment in AUC glucose seen post-exercise post-intervention in the current study is challenging to explain, as fasting glucose and AUC glucose levels were normal the previous day. It must be noted that the participants were still on an LCHF diet when they were exposed to relatively high amounts of glucose during OGTT III and OGTT IV, on two consecutive days. The mechanisms behind the observed reduction in glucose tolerance can only be speculated at this stage, but previous research has shown that the metabolic adaptations that occur in skeletal muscle during an LCHF diet persists despite increased carbohydrate availability (Burke et al., 2002; Stellingwerff et al., 2006; Hawley and Leckey, 2015; Webster et al., 2016). The adaptations include increased rate of fat oxidation and decreased rate of carbohydrate oxidation. Previous studies have shown that high fat feeding decreases carbohydrate oxidation, PDH activation (Bigrigg et al., 2009) and glycogenolysis during exercise (Stellingwerff et al., 2006), which may explain our results. Further, muscle glycogen loading by carbohydrate feeding attenuates AMPK activity both at rest and during exercise (Wojtaszewski et al., 2003). The lower glucose tolerance from OGTT IV indicate that the influence of the glucose load the previous day (OGTT III) had most likely not passed after 24 h when OGTT IV was performed. Therefore, we cannot separate the effect of OGTT III and OGTT IV after the LCHF intervention. The LCHF diet, followed by relatively large loads of glucose on two consecutive days, has possibly resulted in a metabolic inflexibility with a carry-over effect. This might explain the lack of increment in glucose tolerance after exercise during the high-fat diet period.

### Strength and Limitations of the Study

The strength of this study is the daily follow-up and contact with participants, who reported dietary intake, physical exercise and urinary ketones every day. We included only healthy females and OGTTs were performed at the same day in the menstrual cycle. This was done to reduce possible differences due to natural variations seen through the phases of the cycle. All participants adhered closely to the dietary recommendations and compliance was excellent.

The limitations of the present study include the lack of a control group. Additionally, biased reports of diet intake and exercise are known in similar studies. Still, our results from V̇O_2__peak_ tests, weight and body compositions, indicate that the participants did report both diet and exercise accurately. Unfortunately not all food producers include the content of subgroups of fats on the food label, which can lead to miscalculation in the subgroups of fats.

## Conclusion

An LCHF diet with 78 E% fat decreased fasting glucose levels significantly. The diet did not affect fasting insulin or AUC insulin. Free fatty acids, total cholesterol, LDL and LDL/HDL ratio increased significantly. The LCHF diet, followed by two relative large glucose loads on consecutive days has likely caused a metabolic inflexibility, where a bout of exercise was not adequate to counteract the adaptation to the diet. The positive effect of exercise on glucose tolerance seen prior to the intervention was blunted after the intervention. Our data suggest that LCHF diets should not be recommended to healthy, normal weight females, considering the negative changes observed in LDL and total cholesterol, that isolated are factors linked to increased risk for CVD.

## Ethics Statement

This study was approved by the Regional Committee for Medical Research Ethics in Norway (2012/962) and registered in ClinicalTrials.gov, registration number NCT02005224. Subjects gave written informed consent to participate, in accordance with the Declaration of Helsinki (World Medical 2008).

## Author Contributions

TV, JJ, CH, AW, and BN designed the study. TV, BN, and JH carried out the study. NO and PJ analyzed the samples. TV and BN analyzed the data and performed the statistical analysis. JJ, CH, and AW supervised the project. TV wrote the manuscript with help from JJ, CH, and AW. All authors read and approved the final manuscript.

## Conflict of Interest

The authors declare that the research was conducted in the absence of any commercial or financial relationships that could be construed as a potential conflict of interest.

## Footnotes

1 www.somebody.no
